# Genetic architecture of a pollinator shift and its fate in secondary hybrid zones of two *Petunia* species

**DOI:** 10.1186/s12915-023-01561-x

**Published:** 2023-03-20

**Authors:** Marta Binaghi, Korinna Esfeld, Therese Mandel, Loreta B. Freitas, Marius Roesti, Cris Kuhlemeier

**Affiliations:** 1 Institute of Plant Sciences, University of Bern, 3013 Bern, Switzerland; 2 Department of Genetics, Universidade Federal Do Rio Grande Do Sul, Porto Alegre, RS 91501-970 Brazil; 3 Institute of Ecology and Evolution, University of Bern, 3012 Bern, Switzerland

**Keywords:** Adaptive divergence, Biotic selection, Colour, GWAS, Morphology, *Petunia*, Pollination syndrome, Reproductive isolation, Secondary contact

## Abstract

**Background:**

Theory suggests that the genetic architecture of traits under divergent natural selection influences how easily reproductive barriers evolve and are maintained between species. Divergently selected traits with a simple genetic architecture (few loci with major phenotypic effects) should facilitate the establishment and maintenance of reproductive isolation between species that are still connected by some gene flow. While empirical support for this idea appears to be mixed, most studies test the influence of trait architectures on reproductive isolation only indirectly. *Petunia* plant species are, in part, reproductively isolated by their different pollinators. To investigate the genetic causes and consequences of this ecological isolation, we deciphered the genetic architecture of three floral pollination syndrome traits in naturally occurring hybrids between the widespread *Petunia axillaris* and the highly endemic and endangered *P. exserta*.

**Results:**

Using population genetics, Bayesian linear mixed modelling and genome-wide association studies, we found that the three pollination syndrome traits vary in genetic architecture. Few genome regions explain a majority of the variation in flavonol content (defining UV floral colour) and strongly predict the trait value in hybrids irrespective of interspecific admixture in the rest of their genomes. In contrast, variation in pistil exsertion and anthocyanin content (defining visible floral colour) is controlled by many genome-wide loci. Opposite to flavonol content, the genome-wide proportion of admixture between the two species predicts trait values in their hybrids. Finally, the genome regions strongly associated with the traits do not show extreme divergence between individuals representing the two species, suggesting that divergent selection on these genome regions is relatively weak within their contact zones.

**Conclusions:**

Among the traits analysed, those with a more complex genetic architecture are best maintained in association with the species upon their secondary contact. We propose that this maintained genotype–phenotype association is a coincidental consequence of the complex genetic architectures of these traits: some of their many underlying small-effect loci are likely to be coincidentally linked with the actual barrier loci keeping these species partially isolated upon secondary contact. Hence, the genetic architecture of a trait seems to matter for the outcome of hybridization not only then when the trait itself is under selection.

**Supplementary Information:**

The online version contains supplementary material available at 10.1186/s12915-023-01561-x.

## Background

Adaptation to different ecological conditions often drives and maintains reproductive isolation between species despite the opportunity for ongoing gene flow [1–3]. Theory suggests that the efficacy at which ecology-driven reproductive isolation evolves and is maintained depends on the genetic architecture of the traits under divergent natural selection. Reproductive isolation should evolve and be maintained more easily when the traits under divergent selection have a relatively simple genetic architecture with few loci of major effect (or clusters of many loci with small effect) instead of many, genomically interspersed loci with relatively weak effect each [4–6]. This is in part because the genetic architecture of traits — including the number and effect sizes of involved loci as well as possible non-additive (dominance) effects of their alleles — determines the trait distribution in genetically admixed individuals and thus selection against and fitness of naturally-occurring interspecific hybrids (e.g. [7–10]). Understanding the genetic architecture of traits responsible for ecology-driven reproductive isolation is thus key to our understanding of the formation and maintenance of species [11, 12].

An often-used approach to investigate the genetic basis of ecology-driven reproductive isolation is screening the genomes of (partially) reproductively isolated species for regions of exceptional differentiation. While such divergence scans are insightful in describing general patterns of differentiation across the genome, genomic regions of exceptional differentiation can also result from non-adaptive processes or from ecology-unrelated selection and thus only indirectly address the genetic architecture of the actual traits under divergent selection [13–15]. An alternative and more direct approach would be to directly decipher the genetic architecture of the actual traits responsible for ecology-driven reproductive isolation using a forward-genetic approach.

In plants, pollinator specificity is thought to be a major cause for reproductive isolation (for example [16, 17]). Floral traits attracting and rewarding a specific type of pollinator and ensuring pollination efficiency are grouped into sets called “pollination syndromes” [18–21]. Pollination syndromes include visible and UV floral colour traits, as well as morphological traits such as the position of the reproductive organs within flowers [20]. In some plant systems, variation in floral colour was found to be associated with one or few genetic loci of relatively large effect [22], including venation patterning in *Antirrhinum* sp. and petal colour in *Petunia axillaris* and *Petunia inflata* [21, 23–26]. In other cases, however, variation in floral colour appears to have a complex genetic architecture with many loci of relatively small effect each, such as in *Aquilegia* sp. and *P.* *exserta* [27, 28]. Similarly, morphological variation in floral traits related to pollination syndromes seems to have either a relatively simple genetic architecture with few loci of relatively large effect [28, 29] or a complex genetic architecture with many loci of relatively small effect [30, 31]. Overall, these empirical results suggest that few or numerous genetic variants, with relatively large or small phenotypic effect sizes respectively, may underlie adaptation to different pollinators.

Notably, the aforementioned insights on the genetic architecture of pollination syndrome traits in plants come predominantly from QTL mapping — a type of forward-genetic approach that has several shortcomings when trying to understand the genetic architecture of natural trait variation. Most importantly, the genetic and phenotypic diversity investigated by most QTL mapping studies is limited to that of the two parents used to produce the artificial (F2) mapping population [32]. This can bias the estimates of the number and effect sizes of loci underlying trait variation in wild populations [33–35]. Another drawback of QTL mapping is its limited resolution for and even potential bias in estimating the effect size, number, and position of loci underlying trait variation. In particular, the limited recombination of different genomes in one or only few generations as found in experimental mapping populations can affect the results [35–40]. A remedy for these limitations of QTL mapping is provided by genome-wide association study (GWAS), another forward-genetic approach making use of broader variation and more fine-grained recombination in naturally occurring (hybrid) individuals [39].

**Fig. 1:**
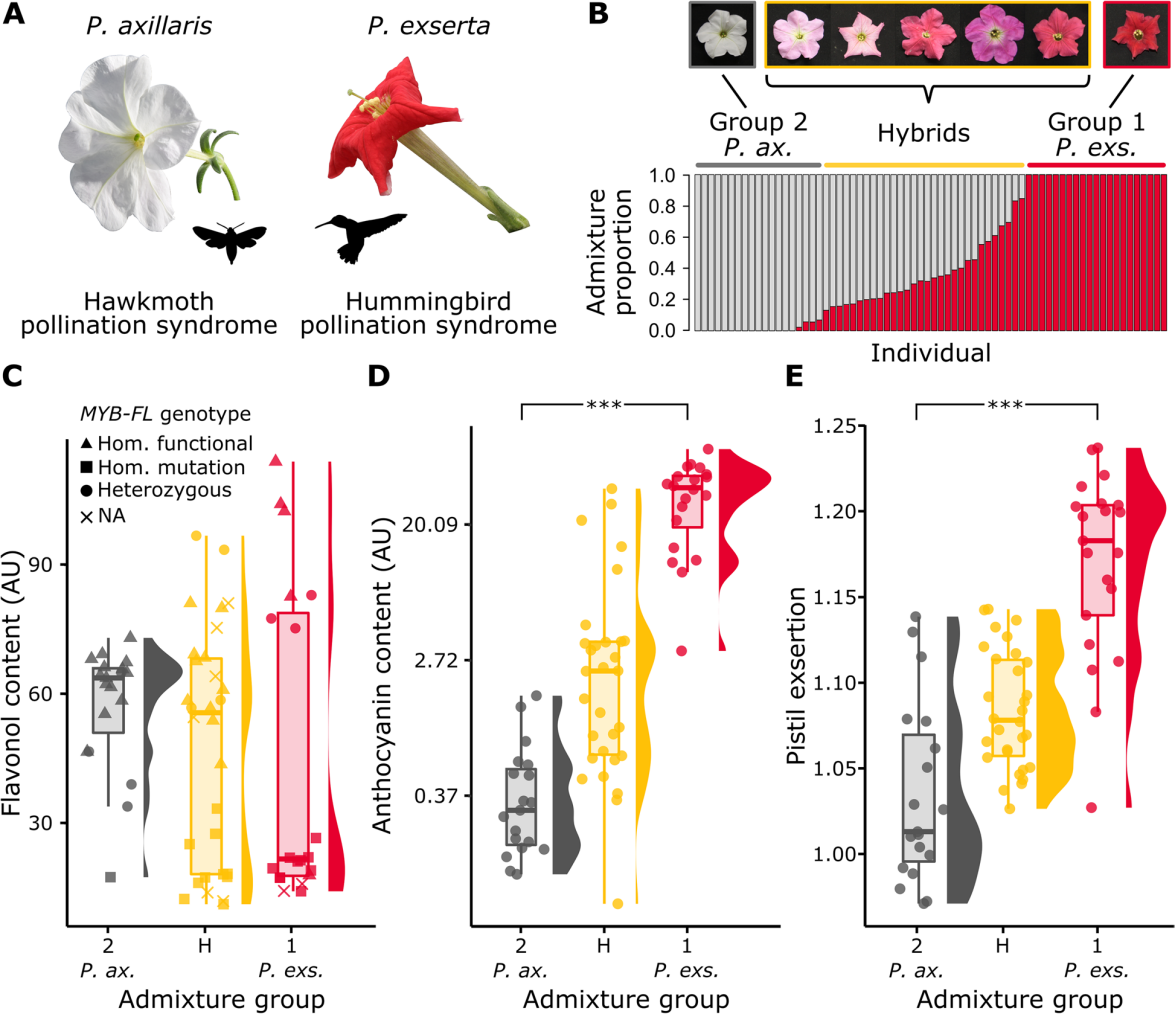
Phenotypic and genetic variation of pure and admixed *P. axillaris* and *P. exserta* individuals. **A** Photos of *P. axillaris* (left) and *P. exserta* (right) flowers. Their pollination syndromes are indicated. **B** Genomic admixture proportion of the analysed individuals for *K* = 2 and photos of some flowers from the admixture groups. The colour diversity in the greenhouse-grown hybrid individuals reflects the diversity found in the wild individuals (Additional file 1: Fig. S3). Groups indicate admixture greater than 0.90 (group 1), admixture lower than 0.10 (group 2), admixture between 0.10 and 0.90 (hybrids). *P. ax.*, *P. axillaris*; *P. exs.*, *P. exserta*. **C**–**E** Phenotypic values for each pollination syndrome trait of all 70 individuals grouped by admixture group as defined in panel B. Points show values of each individual plant, besides boxplots and frequency distributions. Statistically significant differences between group 1 (*n* = 21) and 2 (*n* = 19) are shown with stars (*** indicates a *P* value smaller than 0.001). In panel D, the x-axis is log_10_ scaled for better visualisation of the distributions. In panel C, the symbols indicate the genotype of *MYB-FL* as obtained from CAPS markers genotyping as described in Sheehan et al. (2016). *P. ax.*, *P. axillaris*; *P. exs.*, *P. exserta*

Here, we use GWAS as well as Bayesian linear mixed models and population genetic analyses to decipher the genetic architecture of different pollination syndrome traits and its outcome after hybridization in two plant species exhibiting distinct pollination syndromes [41]. Specifically, we focus on two species of the South American plant genus *Petunia*. *P. axillaris* is hawkmoth-pollinated and has white, UV-absorbent flowers with partially fused petals forming a tube and a limb, and reproductive organs that do not extend outside of the tube (Fig. 1) [41–43]. In contrast, *P.* *exserta* is hummingbird pollinated and has red, UV-reflective flowers, and the reproductive organs extend outside of the petal tube — a typical feature of bird-pollinated flowers (Fig. 1A) [44]. The geographic distributions of the two species overlap partially, but while *P. axillaris* is found in large populations in open habitats such as grasslands, *P. exserta* is an endangered species with few individuals growing in shallow caves on sandstone towers [41].

In the two known cases where *P.* *axillaris* and *P.* *exserta* are not isolated by geography, the species hybridise in secondary contact zones [44]. A recent study estimated this secondary contact of the species to be recent (approximately 920 years ago) and ongoing [45]. Although interspecific hybrids can produce viable offspring and can be found within the hybrid zones, individuals displaying the pure *P.* *axillaris* and *P.* *exserta* phenotypes are found at relatively high frequency [44, 46–48]. This suggests partial reproductive isolation between the species upon secondary contact, likely due to their different pollination syndromes [45, 49–52].

In this study, we investigated the genetic architecture of three floral traits thought to be important in the pollinator-mediated divergence of *P. axillaris* and *P. exserta*, and how these traits and their architectures influence the fate of the pollinator shift between the two species upon secondary contact. Our results shed light on the genetic architecture of pollination syndrome traits, its consequences in hybridization, and its importance for ecological isolation of these species.

## Results

Our study makes use of the two known secondary contact zones of *P. axillaris* and *P. exserta* in southern Brazil, where these species are known to hybridise [44, 47]. We collected seeds from all flowering plants in both contact zones, grew them under standardised conditions in a greenhouse, and sequenced the genomic DNA of 70 of those plants (see Methods section for details). After mapping 150 bp-Illumina reads to the reference genome of *P.* *axillaris* (1.2 Gb total length) and quality filtering, the average genome-wide coverage was 5 × (for more details see Additional file 1: Table S1). The subsequent variant calling and filtering produced the final data set for analysis, containing 4,278,736 biallelic SNPs with a call rate higher or equal to 90% and a minor allele frequency (MAF) higher or equal to 0.05.

Genome-wide variation across all 70 individuals was best grouped into two genetic clusters (Evanno’s method, Additional file 1: Fig. S1), corroborating the results of previous lower-resolution genetic studies on isolated populations and hybrids of *P. axillaris* and *P. exserta* [45, 53]. Across all individuals, the relative assignment into these two genetic clusters correlated strongly with the values along the main axis of a principal component analysis of all SNPs (PC1; Pearson’s correlation =  − 0.99), which explained a high proportion of the total genomic variation (i.e. 19%; Additional file 1: Fig. S2). Furthermore, plants did not separate strongly, if at all, by hybrid zone in any of these analyses, and individuals with varying relative proportions of the two genetic clusters were found in both contact zones (Additional file 1: Fig. S1 and S2). Together with the subsequent phenotypic insights (see next paragraph), these results clearly suggest that the two distinct genetic clusters reflect the different ancestries of the two species [45, 53]. The relative admixture proportion of greater than 90% was thus used as a cut-off to define pure *P. axillaris* and *P. exserta* individuals, while individuals with intermediate admixture proportions were classified as interspecific hybrids [53]. Accordingly, we identified 19 pure *P. axillaris* individuals, 21 pure *P. exserta* individuals, and 30 interspecific hybrids (Fig. 1B, Additional file 1: Fig. S3).

The three key pollination syndrome traits were measured on all 70 individuals and were tested for phenotypic differences between individuals representing the two pure species [41]. (1) Flavonol content, which defines UV absorbance of the petals, did not clearly differ between the two species despite a trend that was consistent with the species definition in which *P. axillaris* is UV absorbent (allowing for hawkmoth attraction in low light conditions) and *P. exserta* is UV reflective (*P* value = 0.18; Fig. 1C and Additional file 1: Table S2). While most *P. exserta* individuals had very low flavonol content as expected, a few individuals had very high flavonol content without any intermediate ones (Fig. 1C). Previously, low flavonol content in *P. exserta* has been associated with a recessive 1-bp deletion leading to a premature stop codon in the transcription factor *MYB-FL* [25]. In line with this, the genotype of *MYB-FL* was a strong predictor of flavonol content in our individuals from interspecific contact zones (Fig. 1C). That is, individuals in the *P. exserta* group with high flavonol content had at least one copy of the functional allele of *MYB-FL*. Intriguingly, flavonol content of those *P. exserta* individuals was higher than the flavonol content of any of the individuals in the *P. axillaris* group. (2) Anthocyanin content was markedly lower in the white *P. axillaris* compared to the red *P. exserta*, in line with its role in defining visible petal colour (*P* value = 2.90 × 10^−11^; Fig. 1D, Additional file 1: Table S2). (3) The exsertion of the pistil, measured as the ratio of the pistil length to the tube length, was clearly and as expected lower in *P. axillaris* than in *P. exserta* (*P* value = 1.13 × 10^−9^; Fig. 1E and Additional file 1: Table S2). The species-specific trends of anthocyanin content and pistil exsertion were also reflected in a much stronger correlation between these two traits compared to flavonol content (Additional file 1: Fig. S4). Moreover, variation in both anthocyanin content and pistil exsertion also correlated strongly with the genomic PC1 as well as with the relative admixture proportion of the two genetic clusters representing the species' different ancestries (see above). In contrast, there was no such correlation for flavonol content (Additional file 1: Table S3 and Fig. S5). To summarise the phenotypic results, while there was no clear difference in flavonol content between individuals from the two different genetic ancestry groups within the two hybrid zones, the clear differences in anthocyanin content and pistil exsertion between these groups were consistent with the phenotypic definition of the two *Petunia* species [41].

To shed light on the genetic architecture of the pollination syndrome shift, we first used a Bayesian sparse linear mixed model (BSLMM) to estimate the genetic architecture for each pollination syndrome trait. In this model, the effect size of every variant is composed of two terms: one of very small (i.e. minor) effect that is common to all variants, and one of larger (i.e. major) effect that is only present for some variants (when reporting the results from this model we will hereafter use the terms of “major” and “minor” effect according to this definition) [54]. The model also provides an estimate of the proportion of phenotypic variance that is explained by the major effect variants (PGE). This represents how much of the variation in the phenotypic trait is explained by major effect variants. Comparing this value for different traits should thus inform on relative differences of their genetic architectures. We found that most variation observed in each trait was explained by the genetic data (PVE above 0.90, Table 1), indicating a high heritability of the observed trait variation under controlled greenhouse conditions. However, the proportion of the total phenotypic variation explained by major effect variants (PGE) varied across the three traits (Table 1). (1) For flavonol content, 84% of the variation was predicted to be controlled by six major effect variants, suggesting a relatively simple genetic architecture with few variants of large overall effect (Table 1). The remaining 16% of the variation in this trait was hence explained by the minor effect of all variants. (2) The genetic architecture for anthocyanin content was predicted to be different, with only 33% of the total trait variation being controlled by as many as 61 variants of major effect (Table 1). This suggests a complex genetic architecture with dozens of “major” variants accounting for only a third of the total trait variation, thus leaving 67% of the trait variation to be controlled by the joint effect of all minor variants in the genome. (3) For pistil exsertion, 51% of the total variation was predicted to be controlled by 42 variants of major effect, while the other half of the trait variation was explained by all genome-wide minor variants (Table 1). The genetic architecture of pistil exsertion thus appears to be intermediate in complexity compared to the architectures of flavonol and anthocyanin content. To summarise the results from BSLMM, the number of predicted variants with substantial phenotypic effect as well as their total contribution to the overall phenotypic variation differs among the three pollination syndrome traits.

**Table 1:** Estimated genetic architectures of traits under divergent pollinator selection using a Bayesian sparse linear mixed model (BSLMM)

Trait	PVE^a^ Mean (SD)	PGE^b^ Mean (SD)	*N* gamma^c^ Mean (SD)
Flavonol content (AU)	0.99 (0.02)	0.84 (0.09)	6 (5)
Anthocyanin content (AU)	0.99 (0.01)	0.33 (0.33)	61 (72)
Pistil exsertion	0.92 (0.07)	0.51 (0.31)	42 (61)

*AU*, absorbance units

^a^Proportion of variance explained by genetic data

^b^Proportion of variance explained by major effect variants

^c^Number of variants with major effect

**Fig. 2:**
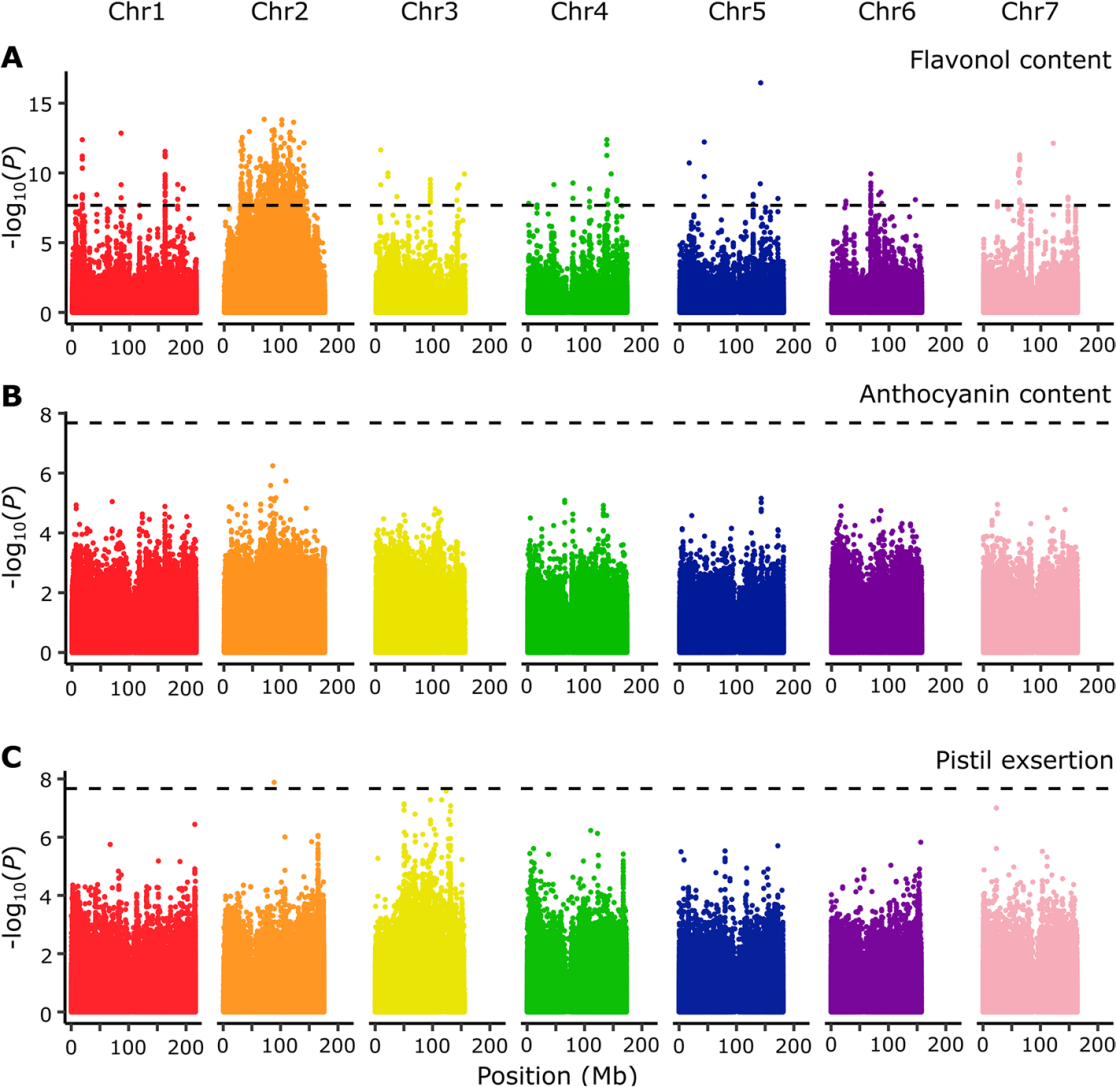
Genomic regions associated with variation in three major pollination syndrome traits. Manhattan plots of the genome-wide association study (GWAS) for **A** flavonol content, **B** anthocyanin content, and **C** pistil exsertion. Dots represent the –log_10_ transformed *P* values of each variant from linear mixed models. The horizontal dashed lines represent the Bonferroni-corrected threshold for *P* = 0.05. Unanchored scaffolds (9 Mb total sequence length) were analysed too but are not shown (notably, none of these scaffolds showed any clear trait association)

We next performed GWAS to validate the BSLMM results and to define genome regions associated with each of the three pollination syndrome traits. We ran one GWAS for each trait using a linear mixed model (LMM) that accounted for population structure and effectively reduced *P* value inflation (Additional file 1: Fig. S6) [55]. (1) Flavonol content was associated with several interspersed genome regions, although a large region of strong association was situated on chromosome 2 (Fig. 2A). This region on chromosome 2 includes the *MYB-FL* gene located at position 111.7 Mb. (2) Anthocyanin content did not reveal clear association peaks in the genome (Fig. 2B), thus fitting the BSLMM prediction that this trait is mostly controlled by very many variants of minor effect (Table 1). Together with the high heritability of this trait (see above), this result suggests that our GWAS had insufficient power to individually detect any of these numerous small-effect variants in the genome. (3) Pistil exsertion showed clearer and sharper association peaks across the genome than anthocyanin content, but only one of those peaks on chromosome 2 proved statistically well supported (Fig. 2C). Although not statistically significant, a relatively large region of chromosome 3 showed an obvious association with variation in the exsertion of the pistil (Fig. 2C). Together, these GWAS results support the predictions from the Bayesian sparse linear mixed model, suggesting that the genetic architecture of the three pollination syndrome traits varies in complexity from relatively simple (flavonol content) to intermediate (pistil exsertion) to complex (anthocyanin content). Since no genome region was clearly associated with more than one trait, the GWAS results also suggest a relatively independent genetic control of each trait.

**Fig. 3:**
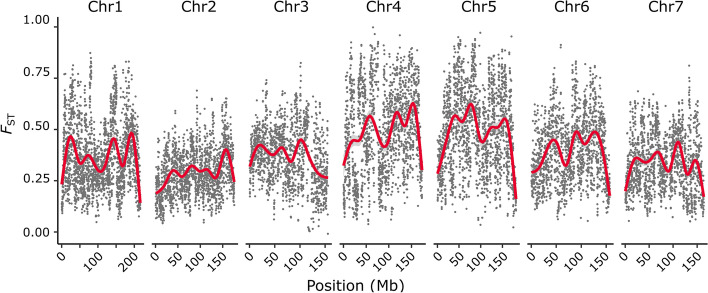
Genetic divergence along the genome between individuals representing the pure species. Each dot represents the average *F*_ST_ of a 200 kb window, with a stepping size of 100 kb. The red lines show smoothed divergence trends along chromosomes as inferred from a generalised additive model. Unanchored scaffolds are not represented as only one of them was longer than the window length of 200 kb. Note that there is no exceptionally strong divergence along chromosomes 2 and 3 on which most large-effect loci were identified by GWAS (see Fig. 2)

Pollinator specificity is thought to be the most important ecological barrier — and the second most important barrier after geography overall — in isolating different *Petunia* species [56]. Genomic regions associated with pollination syndrome traits are therefore expected to show exceptionally strong divergence between *Petunia* species compared to other regions of the genome. To identify genome regions of exceptional interspecific divergence, *F*_ST_ was calculated between individuals representing the pure *P. exserta* and *P. axillaris* species within the hybrid zones. Genome-wide divergence was heterogeneous and high (average *F*_ST_ = 0.27), suggesting strong differentiation between these species despite the possibility for ongoing hybridization. Compared to other chromosomes, chromosomes 4 and 5 showed substantially higher *F*_ST_ (Fig. 3). To test for an overlap between loci associated with pollination syndrome traits and genome regions of exceptional interspecific divergence, the genome windows (200 kb window size) with the highest average *F*_ST_ (i.e. above the 0.95 quantile, *F*_ST_ > 0.73) were compared with genome regions containing significant trait-associated variants as found in the GWAS. The results indicated high-*F*_ST_ genome regions to overlap only with flavonol content associated variants, but a permutation test suggested that these overlaps are likely to have occurred by chance (*P* > 0.05; Additional file 1: Table S4). Because statistically well-supported GWAS variants were only found for flavonol content (except for a single region on chromosome 2 for pistil exsertion; see above), we also tested the association between high-*F*_ST_ windows and the 10 variants with the strongest association for each trait (i.e. lowest *P* value) from the GWAS. While this revealed some overlap between high*-F*_ST_ genome regions and variants for pistil exsertion (besides anthocyanin content), these overlaps again did not prove statistically significant in permutation tests (Additional file 1: Table S5). Overall, we thus conclude that the genome regions associated with pollination traits in this study do not show exceptional divergence between individuals representing pure *P. exserta* and *P. axillaris* species within the hybrid zones. Other regions, such as chromosomes 4 and 5, show much stronger divergence.

## Discussion

In the two contact zones of *P. axillaris* and *P. exserta*, we identified individuals with varying degrees of interspecific admixture based on the analysis of genome-wide variation, ranging from individuals representing the pure species to intermediate interspecific hybrids. Notably, however, more than half of all 70 individuals analysed in this study proved to be (nearly) non-admixed representatives of either one of the two species, suggesting partial reproductive isolation of the species upon secondary contact [47, 48, 53].

Anthocyanin content and pistil exsertion — two important pollination syndrome traits in *Petunia* — were clearly distinct between individuals representing *P. exserta* and *P. axillaris* and presented the values typical of the corresponding species [41]. Flavonol content, however, showed a bimodal distribution across putatively pure *P. exserta* individuals: while most individuals had very low flavonol content as expected for this hummingbird-pollinated plant species, some *P. exserta* individuals showed exceptionally high flavonol content. Upon further examination, all of those high-flavonol *P. exserta* individuals were found to have at least one functional allele of the *MYB-FL* transcription factor which has previously been associated with flavonol content variation using QTL mapping [25]. In line with this result, the genetic architecture of flavonol content was predicted to consist of few major loci explaining most of the observed flavonol variation, and the GWAS identified a strongly-associated genome region on chromosome 2 containing *MYB-FL*. Notably, this genome region extended across a large part of the chromosome. This physically extended signal may be explained by only *MYB-FL* or by *MYB-FL* and further linked variants, as the gene is located within a large genome region of low recombination [43]. The origin of functional *MYB-FL* alleles — previously thought to be typical of *P. axillaris* — in the *P. exserta*-type individuals remains unclear, but possible answers include interspecific introgression, incomplete lineage sorting, or de novo mutation.

The finding of high-flavonol *P. exserta* individuals also influences how we view the importance of this trait in species divergence. As previously argued, flavonol content in the petals defines UV absorption and should therefore affect hawkmoth visitation, whereas UV absorption should be of limited relevance for animals feeding in daylight, such as hummingbirds [25, 57]. It is indeed possible that different pollinators have driven strong differences in UV absorption during the primary divergence of the two *Petunia* species in allopatry. In the secondary contact zones though, the relatively high frequency of UV-absorbent *P. exserta* individuals suggests instead only weak divergent selection on this trait between the species. We speculate that the relatively simple genetic architecture of flavonol content — with one locus of overarching phenotypic effect — facilitated the relatively rapid shift from mostly low-flavonol *P. exserta* in allopatry to high-flavonol *P. exserta* in sympatry with *P. axillaris*.

Not only UV colour, but also the visible colour of flowers is characteristic for different pollination syndromes, and in *Petunia*, the visible colour of petals depends on anthocyanin content [58]. Our model-based prediction of the genetic architecture of anthocyanin content qualitatively matched our GWAS results, both suggesting a highly complex genetic architecture with almost only and thus very many small-effect variants. This complex architecture clearly contrasts with the relatively simple genetic architecture for flavonol content. Interestingly, a previous QTL mapping study in *P. axillaris* × *P. exserta* recombinant inbred lines identified loci on chromosomes 1, 2, 3 and 7 as being responsible for anthocyanin content variation [27]. The functional validation of the candidate genes identified in the study suggested that the shift from white to red colour in these species must have involved multiple genetic changes of subtle effect. Further complexity in this phenotypic trait might also arise from the greater genetic and phenotypic variation found in the hybrid zones.

The model-based prediction for pistil exsertion suggested a genetic architecture that was intermediate in complexity compared to the architectures of flavonol and anthocyanin content. Accordingly, the GWAS identified two regions with strong (albeit statistically non-significant) association with pistil exsertion variation. These regions may thus include some of the causative loci, but surprisingly they do not overlap with the transcription factor EOBII (situated on chromosome 2 at 249.3 Mb) previously shown to affect style length in *P. axillaris* and *P. exserta* [59]. This discrepancy may result from the study systems used; *P. axillaris* and *P. exserta* laboratory accessions used in the study of style length were maintained and inbred for decades. Such inbreeding could cause a loss of the naturally occurring variation, especially in a species such as *P. axillaris* which is known to be relatively variable in the wild [51, 60]. We thus propose that the previously suggested simple genetic architecture for pistil exsertion variation should be revised to be more complex under natural conditions.

Overall, our results indicate that different pollination syndrome traits have different underlying genetic architectures, varying in complexity from few variants of major effect explaining a majority of the phenotypic variation, to several dozens of variants with measurable effects controlling only a third of the phenotypic variation. Especially the complex architecture of anthocyanin content does not clearly follow our classical understanding of visual colour traits being controlled by only few loci of major effect [22, 61]. Instead, in our system, visible colour variation appears to be controlled mostly by very many small-effect loci. The evolutionary path to obtain alleles at so many loci necessary for shifting a trait between two species remains unclear, but it is possible that the relatively long time in allopatry facilitated the evolution of small, gradual trait shifts between our species. Howsoever, a complex genetic architecture with very many loci of small (and additive) effects is certainly not uncommon for quantitative trait variation in nature (e.g. [61–63]).

In theory, fewer loci with relatively large phenotypic effects should build stronger barriers to homogenising gene flow between species, and such simple genetic architectures should also be favoured under divergent selection with gene flow [4, 5]. While the major-effect architecture underlying flavonol content is consistent with this theoretical prediction, the complex genetic architecture underlying anthocyanin content does not match this expectation. In fact, the two traits with the more complex genetic architectures (anthocyanin content and pistil exsertion) showed stronger shifts between individuals representing the two *Petunia* species within the hybrid zones. Accordingly, the interspecific admixture proportion strongly predicted variation in these two traits: an individual with a more *P. axillaris*-like genome had a trait value more typical for *P. axillaris*, and vice versa for *P. exserta.* In contrast, flavonol content was not predicted by an individual’s overall genomic admixture proportion but rather by an individual’s genotype at the major-effect locus *MYB-FL* that appeared to segregate largely independently from the rest of the genome.

With only these phenotypic patterns at hand, it seems obvious to conclude that flavonol content is under weak divergent pollinator selection within the species' contact zones, while the clear shifts in the two other traits could imply strong divergent selection between the species. If so, interspecific differences in the visible colour of petals (anthocyanin content) and in pistil morphology could help isolate *P. axillaris* and *P. exserta* within their contact zones and thus be (partly) responsible for why these species have not fully collapsed into a hybrid swarm despite hundreds of generations of secondary contact [45]. Interestingly, however, the genome regions containing the largest-effect loci for any of the three traits did not show unusually strong differentiation between individuals representing the two pure species within the contact zones. Other genome regions showed much stronger divergence. This raises the intriguing possibility that divergent selection imposed by different pollinators does not reproductively isolate these plant species in their secondary contact zones, but that other ecological factors or ecology-unrelated genetic incompatibilities are more important species barriers.

## Conclusions

We found that the genetic architecture of three typical pollination syndrome traits in *Petunia* vary in complexity from relatively simple to very complex. Theoretically, simple trait architectures with few major-effect loci should facilitate the establishment and maintenance of reproductive isolation between species in the presence of gene flow, including within secondary contact zones. In contrast to this idea, phenotypic differentiation was stronger for traits with complex architectures (many minor-effect loci) between the species in secondary contact zones. However, the genome regions containing the largest-effect loci for any of these traits did not show exceptional divergence between the species. We conclude that while different pollinators might have been important drivers of primary divergence between these plant species in allopatry, pollinator-mediated prezygotic isolation may not be strong and thus does not keep these species apart within contact zones. Genetic incompatibilities or other traits under divergent selection appear to build stronger barriers between these species upon secondary contact. If so, the observed interspecific differences at two of the three pollination syndrome traits might simply be a by-product of the traits' complex genetic architectures. That is, these phenotypic differences may simply be maintained between the species because many of the genome-wide loci underlying these traits are in coincidental linkage with actual barrier loci.

## Methods

### Study populations and plants

This study considers two contact zones of *P.* *axillaris* subsp. *axillaris* and *P.* *exserta* in the Guaritas region of Brazil: contact zone 1 (30° 53′ 48″ S, 53° 25′ 16″ W) and contact zone 2 (30° 50′ 14″ S, 53°30′ 15″ W). These contact zones have been described in much detail before [44, 45, 47, 50, 51, 53, 60, 64]. We collected seeds from all 30 mother plants carrying seed pods at the time of our visit (November 2011). From each seed pod, between 10 and 20 seeds were sown in pots and grown in a greenhouse under standardised conditions to limit environmental effects on the focal traits. To obtain the final set of 70 study individuals, we selected grown plants by maximising trait variation as well as representation of different mother plants (the number of represented mothers was 28, the correspondence between individuals and mother plants is available in Additional file 2).

### Measurement of floral phenotypic traits

From each plant, between 3 and 8 flowers were used for phenotype recording. Flowers were collected 2 days post anthesis and the corolla was cut open longitudinally. The flower was then pinned to a flat surface and photographed. Photos were processed with the software Fiji (ImageJ) to obtain pistil and tube length [65]. Pistil length was measured from the base of the ovary to the top of the stigma. Tube length was obtained by summing the length of the petal tube sections D1 and D2. D1 limits are defined by the petal attachment point at the base of the ovary and the point where the filaments of the stamen detach from the corolla. D2 starts where the filaments detach and end where the corolla bends and constitutes the limb of the petals. Pistil exsertion was calculated as the length of the pistil divided by the length of the tube. The average between flowers was taken to represent the individual plant. After measuring the morphological traits, a disc of 8 mm in diameter was sampled from the corolla limb of the flower. The measurement of the pigments was performed with a spectrophotometer after extraction as described in [25]. We recorded and then averaged 3 to 8 individual flowers for each plant individual to obtain individual trait values. All following analyses were performed on a subset of plants selected among the greenhouse-grown individuals. In order to maximise the phenotypic diversity, we selected individuals displaying different combinations of traits, e.g. a plant with red flowers but a small pistil exsertion, or a plant with white and UV reflective flowers. In order to maximise genetic diversity, we selected plants from most wild mothers we had available (28 out of a total of 30 from which we collected seed pods in the wild). Per plant phenotype measurements are available in Additional file 2. The distributions of phenotype measurements were observed and tested for normality (Shapiro–Wilk test) and rank-transformed when necessary (Additional file 1: Table S2). Difference in phenotype values between the admixture groups was tested with a Wilcoxon rank sum test for flavonol and anthocyanin content and with a Welch *t*-test for pistil exsertion. Correlation between principal genetic components and the phenotypic values was performed with Pearson’s correlation and the results were Bonferroni-corrected. All statistical analyses were performed in R v. 4.1.2, in RStudio 2021.09.1.

### DNA extraction and sequencing

Leaf tissue was collected from each plant and DNA was extracted with a modified CTAB protocol [66]. The DNA was quantified with a fluorometer (Invitrogen Qubit™). Library preparation and sequencing were performed by the Next Generation Sequencing platform of the University of Bern in two batches, one in 2016 and one in 2018. In both batches, the DNA was amplified with illustra™ GenomiPhi™ V2 DNA Amplification Kit. Library preparation followed the TruSeq DNA PCR-free protocol. Sequencing was performed to obtain 150 bp long, paired-end reads, for an estimated coverage of 5 × , on a genome size of 1.2 Gb [67]. The 2016 batch was sequenced on two lanes of an Illumina HiSeq 3000. The 2018 batch was sequenced on two lanes (one chip) of the Illumina NovaSeq. Genotyping of *MYB-FL* was performed as described in Sheehan et al. 2016.

### Read alignment, variant calling and filtering

Raw reads were quality controlled with FastQC version 0.11.7 [68]. Trimming and adapter removal were performed with Trimmomatic version 0.36, with parameters PE, TRAILING:3 SLIDINGWINDOW:4:15 MINLEN:100 [69]. Reads were aligned to the latest reference genome version of *P.* *axillaris* (version 4.03) available on NCBI GenBank under the accession number JANRMM000000000 [70]. Alignment was performed with BWA-MEM 0.7.17 using default parameters, and lanes were merged with samtools 1.10 [71, 72] at bam file stage. A coordinate file was produced to exclude repetitive genome regions from variant calling as well as genome regions with a read coverage higher than 100 in at least one sample. Read alignment metrics are available in Additional file 3. GATK 4.1.3.0 and the tools included in it [73] were used to mark duplicated reads and to perform variant calling in GVCF mode with default parameters. The obtained variants were then hard filtered following the GATK best practices for organisms that lack panels of high-quality variants. Before applying the GATK-suggested thresholds, the quality parameters were extracted for SNPs and their distribution was observed to confirm that filter thresholds were appropriate. After quality filtering, variants were then filtered to keep only SNPs at positions with 90% or higher call rate, and a minor allele frequency of 0.05 or higher. These filters produced a set of 4,278,736 variants.

### Population genomics and GWAS analyses

Population genomic analyses (admixture, PCA and *F*_ST_) were performed on genotype likelihoods rather than on called genotypes, to account for the uncertainty introduced by the low coverage on the genotype calls [74]. Genotype likelihoods were calculated from the PL field in the vcf file produced by GATK with a custom Perl script available at https://github.com/kuhlemeier-lab/petunia_hybrid_pollinator_genetics. The genotype likelihoods were then used to perform an admixture analysis with NgsAdmix available in ANGSD version 0.933 [75, 76]. *K* from 1 to 8 were tested, and the analysis was repeated 10 times. The run with the best likelihood was chosen for each *K*. The most likely *K* was calculated using Evanno’s method [77] implemented in an R script. PCA analysis was performed with pcangsd version 1.02 [74].

The GWAS and the predictions of the genomic architecture for each trait were performed with GEMMA version 0.98.4 [55]. To do so, the vcf file was converted to the bimbam format with a modified version of a Perl script by Victor Soria-Carrasco available at https://github.com/visoca/popgenomworkshop-gwas_gemma. The phenotype files were formatted according to GEMMA guidelines in R. To estimate genetic architectures, we used the Bayesian sparse linear mixed model (BSLMM). Its application allows for the correct modelling of traits controlled by few variants of major effect and additional small effect variants, as well as traits controlled only by numerous small effect variants [54]. We used options -bslmm 1 (fits a linear BSLMM using MCMC), -w 50,000,000 (discarded burn-in iterations), -s 200,000,000 (saved sampling iterations), -pval 4, -hwe 0.001, -miss 0, -maf 0 [54]. The mean and SD of each hyperparameter were calculated in R. For the genome-wide association analysis, we used the linear and linear mixed models available in GEMMA to test the association of each variant to the phenotype. We used the variant dataset with MAF > 0.05 and call rate > 0.90, including 4,278,736 variants distributed along the genome as input file. Both models test each single variant for the alternative hypothesis of having a significant effect on the phenotype, while the null hypothesis is that the variant has no effect on the phenotype. The presence of population structure can strongly affect the results of a GWAS, but can be accounted for by including the kinship matrix (which holds information on the genetic structure of the population) as a random effect in a linear mixed model [78]. We thus computed the kinship matrix with the function -gk 1 in GEMMA and tested a linear model which does not account for population structure, and a linear mixed model which instead does. We verified that the linear mixed model reduced the inflation of the *P* values (Additional file 1 Fig. S6), hence we continued the analyses with this model. We note that the small sample size of our study limits the power of the association mapping; in particular, the statistical significance of loci of small to medium effect is hampered by the strict threshold imposed by the high number of variants considered. The results displayed for the linear mixed model were obtained with option -lmm 4, after kinship matrix estimation with option -gk 1, -hwe 0.001 -miss 0 -maf 0. The likelihood ratio test was considered for significance.* P* values were corrected for multiple testing by dividing the canonical 0.05 threshold by the number of variants tested. Manhattan plots were produced in R, and display the -log_10_-transformed *P* values. In the plot, a transformed *P* value above the threshold indicates that the variant is significantly associated with the phenotype. Q-Q plots were produced in R. *F*_ST_ between admixture groups was calculated with ANGSD using the unfolded spectra, and averaged over 200 kb windows, with stepping size 100 kb. A permutation test was used to evaluate the overlap between regions of high *F*_ST_ and regions identified in the GWAS. For this, the region surrounding each significant variant in the GWAS was selected using a window of 20 kb (10 kb on each side of the variant position). To test overlap in traits that had no or few sites passing the Bonferroni-corrected threshold, we selected the ten sites with the lowest *P* value in each trait, and used the 20 kb surrounding region. In addition, we tested all the regions just described for overlap with chromosome-wise high *F*_ST_ regions. For this, we considered one chromosome at a time, selected the regions with a *F*_ST_ above the 95th quantile of the chromosome, and calculated overlap with the GWAS variant regions of that chromosome (which are in any case, genome-wide). The overlap test was performed with the R library regioneR on a significance threshold for overlap of 0.05 [79].

Resource-demanding computations were performed on UBELIX (http://www.id.unibe.ch/hpc), the HPC cluster at the University of Bern. Statistical data analyses and plotting were performed in R [80, 81].

Complete scripts used in the analyses are available at https://github.com/kuhlemeier-lab/petunia_hybrid_pollinator_genetics.

## Supplementary Information

**Additional file 1: Table S1.** Average read coverage per individual on genome and gene regions. **Table S2. **Normality of the trait distribution and statistical difference between phenotype of the pure species individuals. **Table S3. **Correlation between phenotype and genetic principal components. Pearson’s correlation, with Bonferroni correction. **Table S4. **Overlap between loci with statistically significant association to phenotypic traits and genome regions of high interspecific differentiation, as calculated by *F*_ST_. **Table S5. **Overlap between the 10 loci with the strongest association to each phenotypic trait with genome regions of high interspecific differentiation, as calculated by *F*_ST_. **Figure S1. **Evanno’s choice of K and individual admixture proportions for K = 2 and K = 3. **Figure S2. **Major axes of a PCA of genome-wide variation of *P. axillaris* and *P. exserta* individuals and their hybrids from natural contact zones. **Figure S3. **Broad variation in visible floral colour in wild individuals growing in the hybrid zones. **Figure S4. **Phenotypic trait correlations. **Figure S5. **Association between interspecific admixture proportion and trait values. **Figure S6. **Q-Q plots of the GWAS *P* values.

**Additional file 2. **Phenotype measurement data. The table includes the phenotypic values of each plant. The details on the headers of the measurement files are found in the github repository, at https://github.com/Kuhlemeier-lab/Petunia_hybrid_pollinator_genetics/blob/main/data/phenotype_header_info.csv.

**Additional file 3. **Read and alignment metrics. Table showing the read pair numbers and genome coverage of each DNAseq library.

## Data Availability

The phenotype datasets supporting the conclusions of this article are included within the article and its additional files. The raw reads supporting the conclusions of this article are available in the NCBI SRA repository, under the BioProject accessions PRJNA522653 (2016 batch, https://identifiers.org/bioproject:PRJNA522653) [82] and PRJNA706535 (2018 batch, https://identifiers.org/bioproject:PRJNA706535) [83]. Phenotype measurements, scripts and parameters used in the analyses are available on GitHub, at https://github.com/kuhlemeier-lab/petunia_hybrid_pollinator_genetics [84].

## Abbreviations

AU: Absorbance units
BSLMM: Bayesian sparse linear mixed model
EOBI: Emission of benzenoids I
EOBII: Emission of benzenoids II
GWAS: Genome-wide association study
MYB-FL: R2R3-MYB transcription factor
PC: Principal component
PCA: Principal component analysis
PGE: Proportion of genetic variance
PVE: Proportion of variance in phenotypes
QTL: Quantitative trait locus
UV: Ultra-violet
