# Supplementary material for: Genetic architecture of a pollinator shift and its fate in secondary hybrid zones of two Petunia species
Source: BMC Biol. 2023 Mar 20;21:58. doi: 10.1186/s12915-023-01561-x (PMC10029178; doi:10.1186/s12915-023-01561-x)
Supplement: Supplementary file 1 — Additional file 1: Table S1. Average read coverage per individual on genome and gene regions. Table S2. Normality of the trait distribution and statistical difference between phenotype of the pure species individuals. Table S3. Correlation between phenotype and genetic principal components. Pearson’s correlation, with Bonferroni correction. Table S4. Overlap between loci with statistically significant association to phenotypic traits and genome regions of high interspecific differentiation, as calculated by FST. Table S5. Overlap between the 10 loci with the strongest association to each phenotypic trait with genome regions of high interspecific differentiation, as calculated by FST. Figure S1. Evanno’s choice of K and individual admixture proportions for K = 2 and K = 3. Figure S2. Major axes of a PCA of genome-wide variation of P. axillaris and P. exserta individuals and their hybrids from natural contact zones. Figure S3. Broad variation in visible floral colour in wild individuals growing in the hybrid zones. Figure S4. Phenotypic trait correlations. Figure S5. Association between interspecific admixture proportion and trait values. Figure S6. Q-Q plots of the GWAS P values. [file 12915_2023_1561_MOESM1_ESM.pdf]

Additional file 1: Additional tables and figures

Tab. S1 Average read coverage per individual on genome and gene regions

Metric	Value
Average coverage genome	5.19
Average coverage gene regions	6.20
Proportion of genome covered by 1 read or more	0.79
Proportion of genome covered by 5 reads or more	0.46
Proportion of gene regions covered by 1 read or more	0.87
Proportion of gene regions covered by 5 reads or more	0.56

Tab. S2 Normality of the trait distribution and statistical difference between phenotype of the pure species individuals

Trait	Normality test[a]		Difference between groups		
	W	*P* value	Statistic	Statistic value	*P* value
Flavonol content	0.92074	3.15×10^{-4}	W[b]	239	0.18
Anthocyanin content	0.71978	3.59×10^{-10}	W[b]	380	2.90×10^{-11}
Pistil exsertion	0.97366	0.15	t[c]	- 8.018	1.13×10^{-9}

[a] Shapiro-Wilk normality test

[b] Wilcoxon rank sum test

[c] Welch *t* test

Tab. S3 Correlation between phenotype and genetic principal components. Pearson's correlation, with Bonferroni correction.

Phenotypic trait	PC (percent variance explained)	Pearson r^2	*P* value	Bonferroni *P* value	Bonferroni significance
Antho. cont.	PC1 (18.83)	-0.83	2.4e-18	1.2e-16	**
Antho. cont.	PC2 (9.79)	0.03	8.1e-01	4.1e+01	n.s.
Antho. cont.	PC3 (7.39)	0.09	4.5e-01	2.2e+01	n.s.
Antho. cont.	PC4 (6.44)	-0.13	3.0e-01	1.5e+01	n.s.
Antho. cont.	PC5 (4.97)	-0.22	7.0e-02	3.5	n.s.
Antho. cont.	PC6 (4.42)	0.02	8.8e-01	4.4e+01	n.s.
Antho. cont.	PC7 (3.68)	0.00	9.7e-01	4.8e+01	n.s.
Antho. cont.	PC8 (3.29)	-0.01	9.6e-01	4.8e+01	n.s.
Antho. cont.	PC9 (2.61)	0.10	4.1e-01	2.0e+01	n.s.
Antho. cont.	PC10 (2.48)	0.04	7.2e-01	3.6e+01	n.s.
Flav. cont.	PC1 (18.83)	0.17	1.5e-01	7.6	n.s.
Flav. cont.	PC2 (9.79)	-0.42	3.7e-04	1.8e-02	*
Flav. cont.	PC3 (7.39)	-0.10	4.0e-01	2.0e+01	n.s.
Flav. cont.	PC4 (6.44)	-0.28	1.8e-02	9.0e-01	n.s.
Flav. cont.	PC5 (4.97)	-0.14	2.6e-01	1.3e+01	n.s.
Flav. cont.	PC6 (4.42)	0.45	1.2e-04	5.9e-03	**
Flav. cont.	PC7 (3.68)	-0.18	1.4e-01	6.9	n.s.
Flav. cont.	PC8 (3.29)	-0.07	5.9e-01	2.9e+01	n.s.
Flav. cont.	PC9 (2.61)	0.02	8.8e-01	4.4e+01	n.s.
Flav. cont.	PC10 (2.48)	-0.24	5.1e-02	2.6	n.s.
Pistil exsertion	PC1 (18.83)	-0.79	5.3e-16	2.6e-14	**
Pistil exsertion	PC2 (9.79)	-0.14	2.4e-01	1.2e+01	n.s.
Pistil exsertion	PC3 (7.39)	-0.15	2.1e-01	1.1e+01	n.s.
Pistil exsertion	PC4 (6.44)	0.01	9.2e-01	4.6e+01	n.s.
Pistil exsertion	PC5 (4.97)	0.09	4.5e-01	2.2e+01	n.s.
Pistil exsertion	PC6 (4.42)	-0.08	5.2e-01	2.6e+01	n.s.
Pistil exsertion	PC7 (3.68)	0.08	5.3e-01	2.7e+01	n.s.
Pistil exsertion	PC8 (3.29)	0.08	5.1e-01	2.6e+01	n.s.
Pistil exsertion	PC9 (2.61)	-0.13	2.9e-01	1.4e+01	n.s.
Pistil exsertion	PC10 (2.48)	0.13	2.7e-01	1.3e+01	n.s.

Tab. S4 Overlap between loci with statistically significant association to phenotypic traits and genome regions of high interspecific differentiation, as calculated by F_{ST}. Statistical significance of the overlap was calculated with a permutation test.

Trait	Genomic region[a]	F_{ST} 0.95 quantile[b]	N F_{ST}[c]	N GWAS[d]	N overlapping[e]	*P* value permutation
Flavonol content	Genome	0.73	598	835	7	1
	Chr1	0.66	107	44	0	
	Chr2	0.48	87	709	43	0.88
	Chr3	0.60	75	19	0	
	Chr4	0.82	84	20	0	
	Chr5	0.82	89	10	0	
	Chr6	0.68	78	18	6	0.10
	Chr7	0.57	80	15	2	0.38
Anthocyanin content	Genome	0.73	598	0	0	
	Chr1	0.66	107	0	0	
	Chr2	0.48	87	0	0	
	Chr3	0.60	75	0	0	
	Chr4	0.82	84	0	0	
	Chr5	0.82	89	0	0	
	Chr6	0.68	78	0	0	
	Chr7	0.57	80	0	0	
Pistil exsertion	Genome	0.73	598	1	0	
	Chr1	0.66	107	0	0	
	Chr2	0.48	87	1	0	
	Chr3	0.60	75	0	0	
	Chr4	0.82	84	0	0	
	Chr5	0.82	89	0	0	
	Chr6	0.68	78	0	0	
	Chr7	0.57	80	0	0	

[a] Genomic region considered to calculate the value corresponding to the 0.95 quantile of the F_{ST}
[b] Value of the 0.95 quantile of the F_{ST} for the genomic region considered
[c] Number of regions with an F_{ST} higher than the 0.95 quantile
[d] Number of sites with statistically significant association to the phenotypic trait in the genomic region considered
[e] Number of regions that overlap between the two sets

Tab. S5 Overlap between the 10 loci with the strongest association to each phenotypic trait with genome regions of high interspecific differentiation, as calculated by F_{ST}. Statistical significance of the overlap was calculated with a permutation test.

Trait	Genomic region[a]	F_{ST} 0.95 quantile[b]	N F_{ST}[c]	N GWAS[d]	N overlapping[e]	*P* value permutation
Flavonol content	Genome	0.73	598	10	0	
	Chr1	0.66	107	0	0	
	Chr2	0.48	87	9	0	
	Chr3	0.60	75	0	0	
	Chr4	0.82	84	0	0	
	Chr5	0.82	89	1	0	
	Chr6	0.68	78	0	0	
	Chr7	0.57	80	0	0	
Anthocyanin content	Genome	0.73	598	10	1	0.58
	Chr1	0.66	107	1	0	
	Chr2	0.48	87	7	0	
	Chr3	0.60	75	0	0	
	Chr4	0.82	84	1	0	
	Chr5	0.82	89	1	0	
	Chr6	0.68	78	0	0	
	Chr7	0.57	80	0	0	
Pistil exsertion	Genome	0.73	598	10	0	
	Chr1	0.66	107	0	0	
	Chr2	0.48	87	1	0	
	Chr3	0.60	75	8	4	0.05
	Chr4	0.82	84	0	0	
	Chr5	0.82	89	0	0	
	Chr6	0.68	78	0	0	
	Chr7	0.57	80	1	0	

[a] Genomic region considered to calculate the value corresponding to the 0.95 quantile of the F_{ST}

[b] Value of the 0.95 quantile of the F_{ST} for the genomic region considered

[c] Number of regions with an F_{ST} higher than the 0.95 quantile

[d] Number of sites with an association to the phenotypic trait among the strongest ten, in the genomic region considered (across the genome they are necessarily ten).

[e] Number of regions that overlap between the two sets

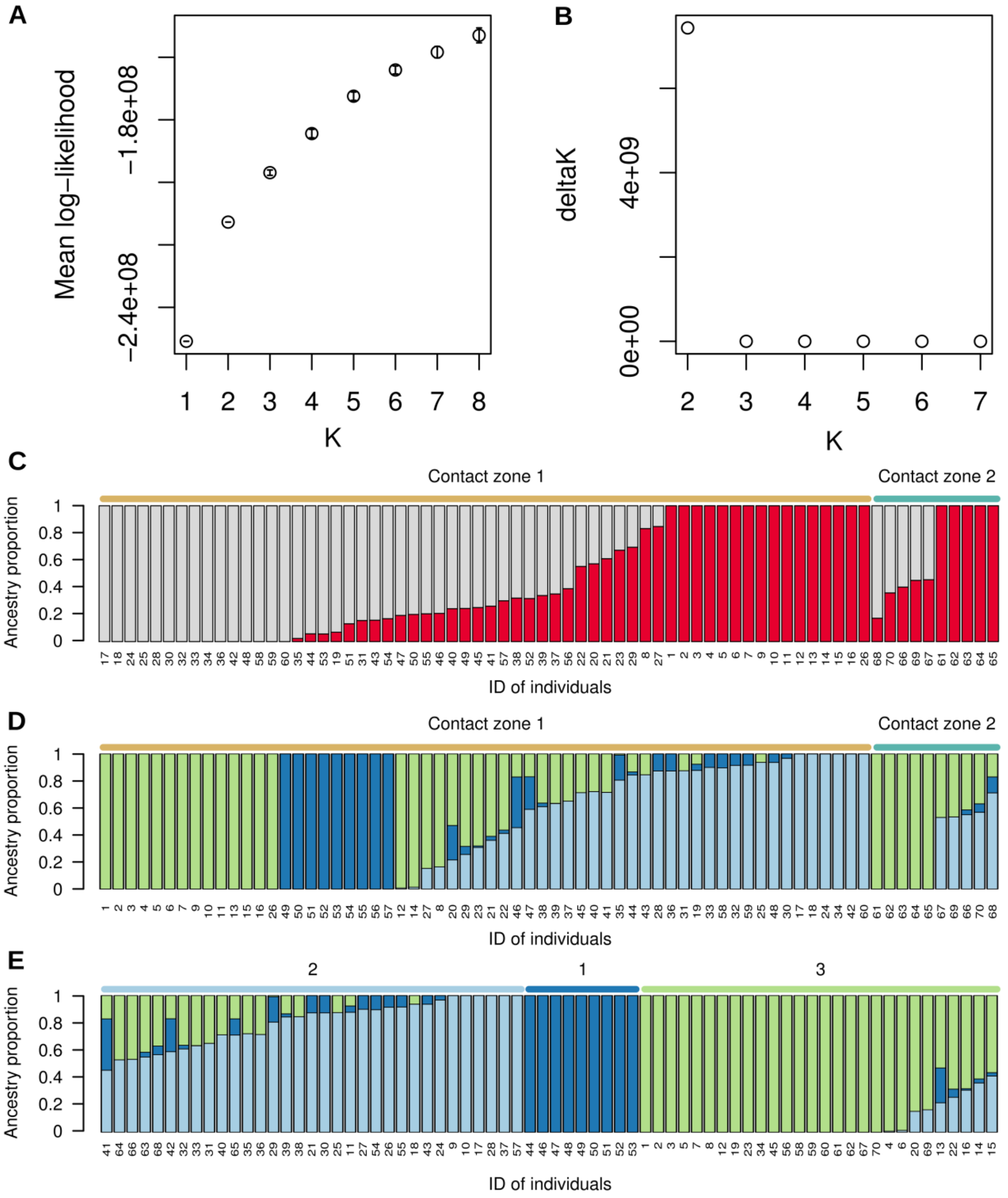

Fig. S1 Evanno's choice of *K* and individual admixture proportions for *K* = 2 and *K* = 3. A) Log-likelihood mean and standard deviation of each *K* tested in NGSADMIX. B) Delta of the log-likelihoods. C) Admixture proportions for *K* = 2, with individuals sorted by contact zone of origin. D, E) Admixture proportions for *K* = 3, with individuals sorted by contact zone of origin and by major ancestry proportion. Note that there is no clear clustering of the individuals according to their contact zone of origin in C) and D).

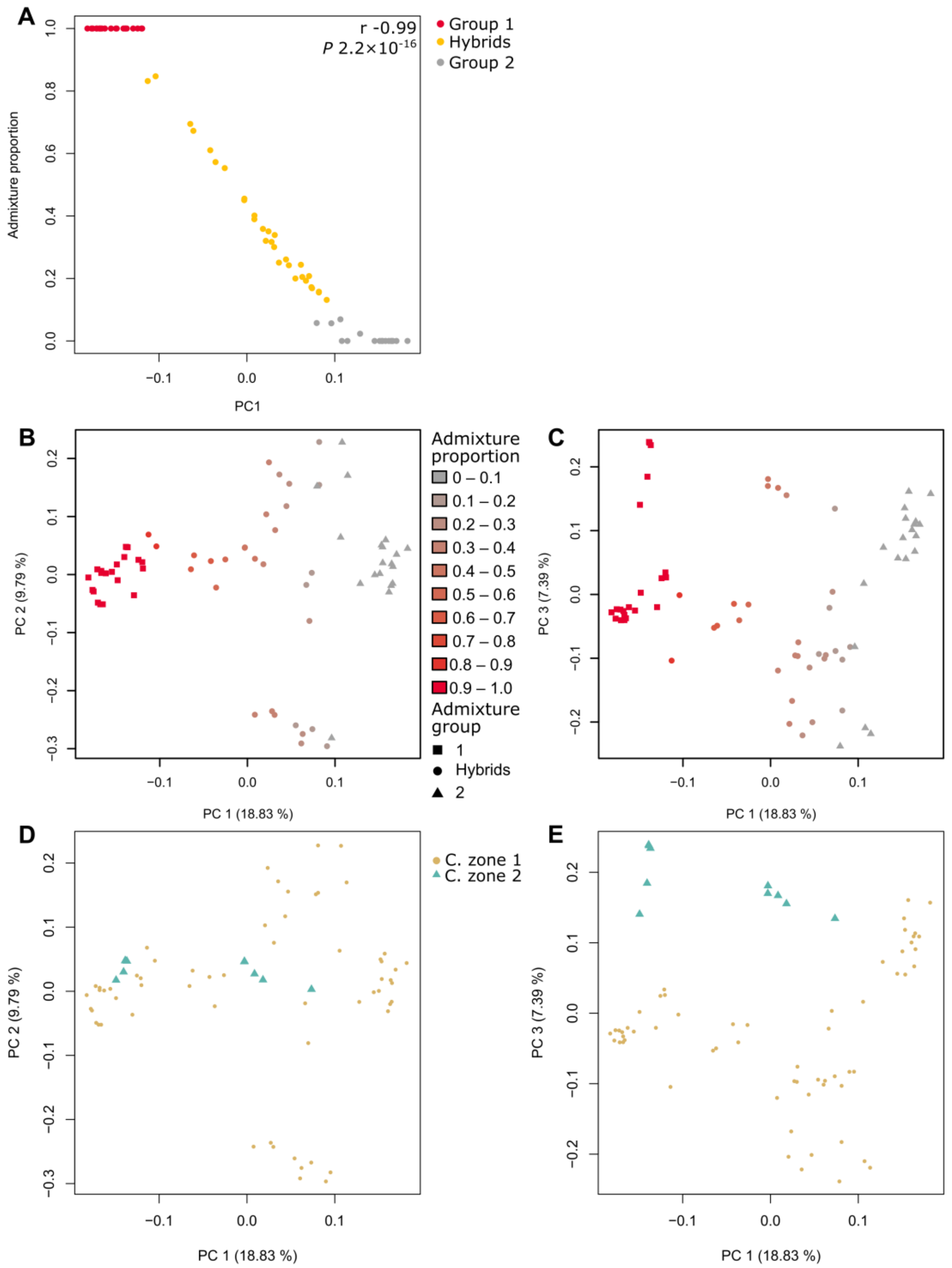

Fig. S2 Major axes of a PCA of genome-wide variation of *P. axillaris* and *P. exserta* individuals and their hybrids from natural contact zones. A) Relative admixture proportion of the two distinct genetic clusters against genomic PC1 for all individuals. Colours represent the admixture groups as defined in Fig. 1. Indicated is also the strength and statistical significance of the Pearson's correlation. Plots of the B) first against second, and C) first against third genomic PC axis across all individuals. The colour represents the individual admixture proportion assigned for $K = 2$, the symbol represents the admixture groups as defined in Fig. 1. D) and E) are identical to B) and C) but colours here represent the different contact zones of the individuals. Note that in B) and D), PC2 shows an axis of variation mostly present in individuals of group 2, which could reflect *P. axillaris* greater genomic diversity. In panel E, PC1 and PC3 show that individuals from contact zone 2 do not overlap with individuals from contact zone 1, possibly indicating that the two contact zones have some genomic differences.

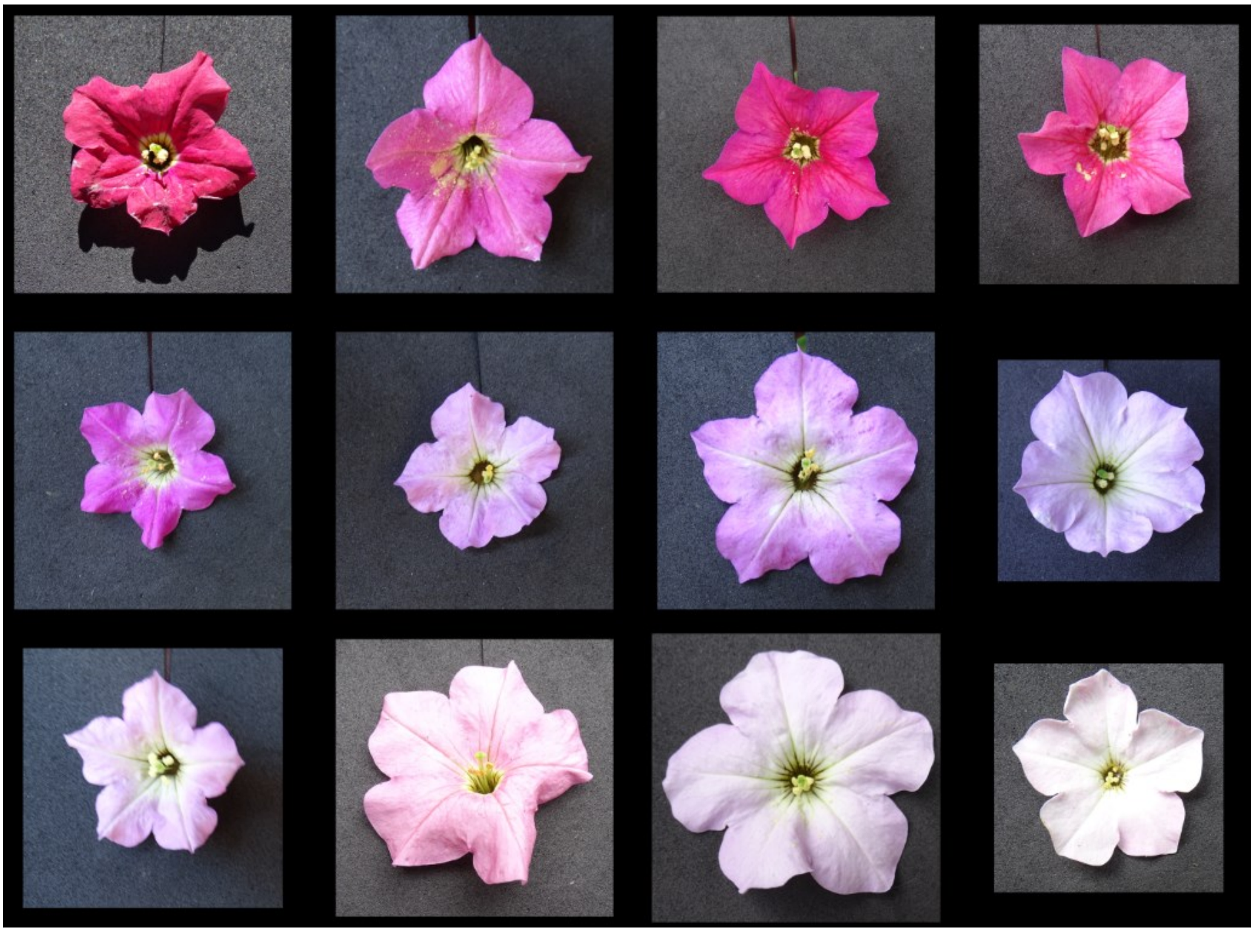

Fig. S3 Broad variation in visible floral colour in wild individuals growing in the hybrid zones. Photos of flowers from individuals growing in the hybrid zones of *P. axillaris* and *P. exserta*. The colour spectrum in the greenhouse-grown plants (Fig. 1) reflects the variation in wild. Photos courtesy of Marcelo Costa Teixeira (Federal University of Rio Grande do Sul, Brazil).

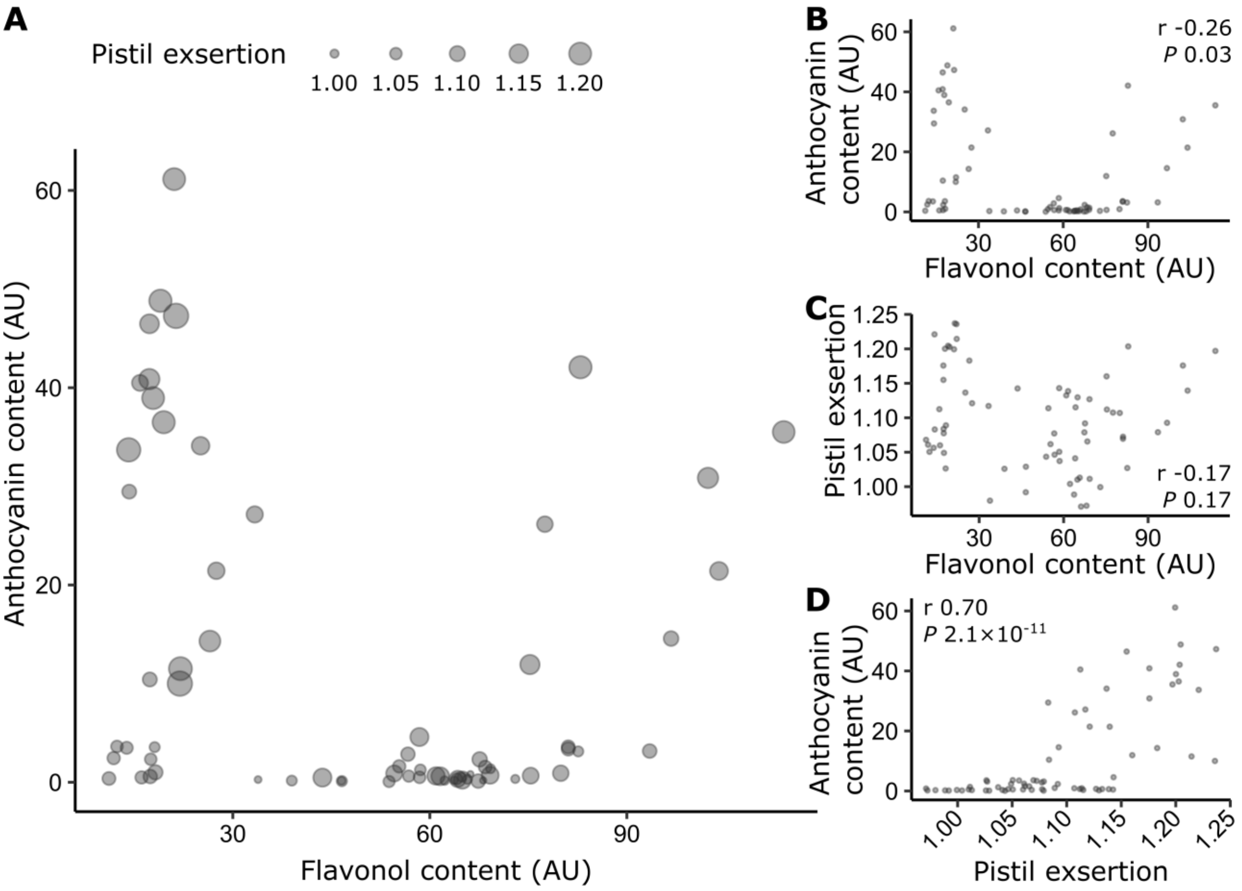

Fig. S4 Phenotypic trait correlations. A) Flavonol content plotted against anthocyanin content. Each dot represents an individual, the radius of the dots is proportional to the pistil exsertion value. B-D) Scatter plots of pairwise trait comparison of the three pollination syndrome traits measured. r and *P* indicate the strength and statistical significance of the Pearson's correlation.

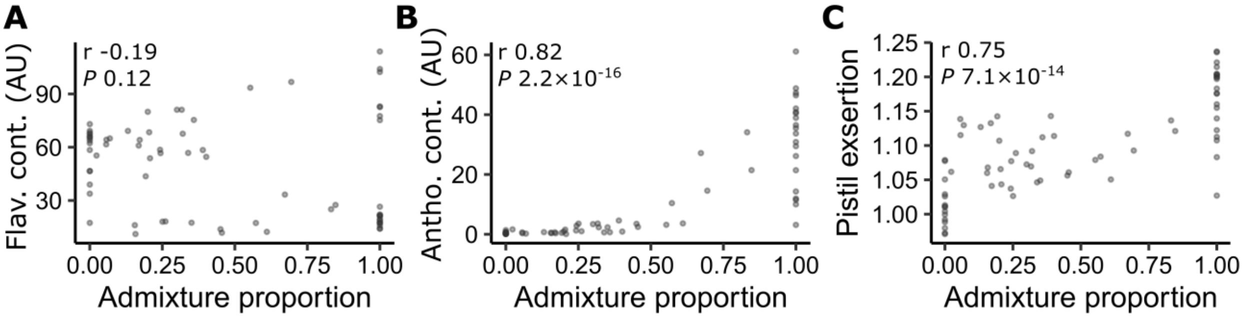

Fig. S5 Association between interspecific admixture proportion and trait values. Scatter plots of each trait against the relative admixture proportion. Each dot is an individual. r and *P* indicate the strength and statistical significance of the Pearson's correlation.

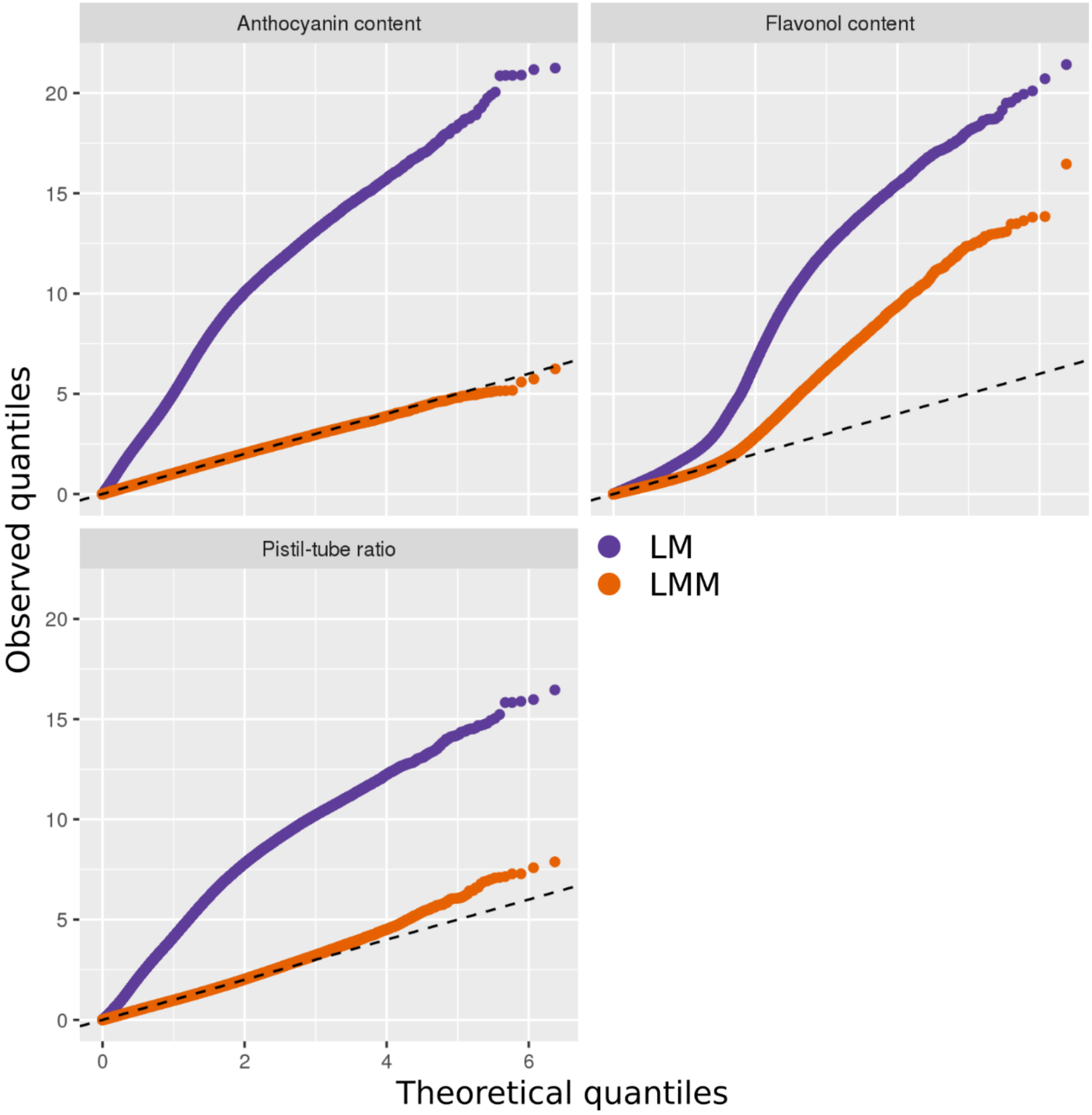

Fig. S6 Q-Q plots of the GWAS *P* values. Quantile-quantile plots of the theoretical and observed *P* values obtained with different models in the GWAS analysis. LM linear model, LMM linear mixed model.
